# Design of a Wireless Sensor Module for Monitoring Conductor Galloping of Transmission Lines

**DOI:** 10.3390/s16101657

**Published:** 2016-10-09

**Authors:** Xinbo Huang, Long Zhao, Guimin Chen

**Affiliations:** 1 School of Electronics Information, Xi’an Polytechnic University, Xi’an 710048, China; 2 School of Electro-Mechanical Engineering, Xidian University, Xi’an 710070, China; zhaolong@xpu.edu.cn (L.Z.); gmchen@mail.xidian.edu.cn (G.C.)

**Keywords:** wireless sensor module, transmission lines, conductor galloping, inertial measurement unit

## Abstract

Conductor galloping may cause flashovers and even tower collapses. The available conductor galloping monitoring methods often employ acceleration sensors to measure the conductor translations without considering the conductor twist. In this paper, a new sensor for monitoring conductor galloping of transmission lines based on an inertial measurement unit and wireless communication is proposed. An inertial measurement unit is used for collecting the accelerations and angular rates of a conductor, which are further transformed into the corresponding geographic coordinate frame using a quaternion transformation to reconstruct the galloping of the conductor. Both the hardware design and the software design are described in details. The corresponding test platforms are established, and the experiments show the feasibility and accuracy of the proposed monitoring sensor. The field operation of the proposed sensor in a conductor spanning 734 m also shows its effectiveness.

## 1. Introduction

**Figure 1:**
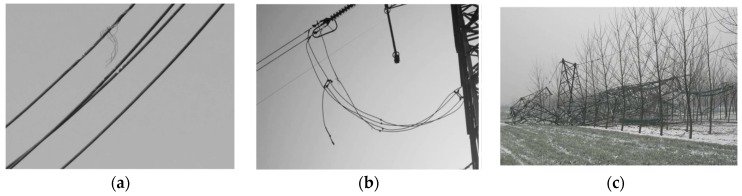
Damages caused by conductor galloping: (**a**) depiction of the damages caused by conductor broken stocks; (**b**) depiction of the damages caused by tension fitting fracture, and suspension clamp breakage; (**c**) depiction of the damages caused by tower collapses.

Conductor galloping involving various voltage levels of transmission lines occurs frequently around the world [1,2,3,4]. It is considered as a self-excited vibration of low frequency and large amplitude caused by non-uniform icing and strong winds. The damages produced by conductor galloping may manifest in many ways, e.g., deformation of tower cross-arms, flashovers between conductors, and even tower collapse [4,5,6,7], as shown in Figure 1.

The causes and the characteristics of conductor galloping are complicated due to the complexities related to system parameters, external environment parameters and various stochastic factors, which bring about many challenges to the research on conductor galloping. To date, much work has been focused on galloping prevention. For example, suspension clamps are often mounted on the transmission lines to prevent galloping. However, while this may be effective for some transmission lines, it may not work for others. Therefore, accurate simulation of the conductor galloping condition is necessary for the design of a satisfactory anti-galloping device. Dynamic tension variation is the most common feature of conductor galloping [8]. Some people have adopted the average method to get an analytical solution of galloping amplitude, and they found that the damping ratio, wind velocity and mass ratio were the three most important parameters of the conductor galloping model [9]. The Hamilton principle was also used for analyzing the galloping vertical amplitude and torsional angle with different influencing factors. It was shown that the most significant factors included wind velocity, flow density, span length, damping ratio, and initial tension [10].

Research on conductor galloping monitoring technology aims to obtain the key data about galloping for scientific research on galloping mechanisms and its prevention. In the past decades, with continuous improvement of theoretical galloping models, sensor technology and communication technology, the monitoring technology of conductor galloping is developing rapidly, e.g., image/video surveillance [4,5,6,7,11], and acceleration sensor monitoring methods [7,11,12]. Although the image/video surveillance method is more intuitive, the conductors are often too long to be observed using video or image, especially since the device may swing when conductors vibrate sharply. The acceleration sensor monitoring method may cause the output data to not be in the same reference coordinates due to the inevitable conductor torsion, which brings about a large deviation from the actual movement. Besides, the maximum amplitude estimated by mathematical models has been put forward but it still needs to be verified by examples [13]. A monitoring scheme based on the fiber Bragg grating sensor was also proposed to monitor conductor galloping [14], but it suffered from some difficulties in practice. Meanwhile, the Micro-Electro-Mechanical-Systems technology is attracting more and more attention and provides smaller and cheaper sensors which can measure physical quantities such as acceleration and angular velocity, allowing inertial sensors to be widely used in motion analysis and tracking applications [15,16]. Using inertial measurement units to monitor galloping can avoid errors caused by conductor twist [17]. However, reference [17] did not mention how to deal with trend items which often appear and directly influence the measurement results. 

Aiming at monitoring the motion state of conductor galloping and using it as a basic unit of an integrated online-monitoring system for transmission lines, a wireless sensor is proposed in this paper, which utilizes an inertial measurement unit to collect accelerations and angular rates of conductor motion and then reconstructs the conductor galloping in 3D space through a series of algorithms. The methods for calculating accelerations, velocity and displacement are deduced in details. During the derivation procedure, the trend items of the conductor galloping have been considered, and the whole conductor galloping trajectory is obtained using the Bezier curve fitting method for the first time. The corresponding test platforms are set up and a series of experiments are carried out to evaluate its feasibility and accuracy. Moreover, it was put into operation in a conductor span of 734 m, and the results show that the proposed wireless sensor module for monitoring conductor galloping of transmission lines is both feasible and effective.

**Figure 2:**
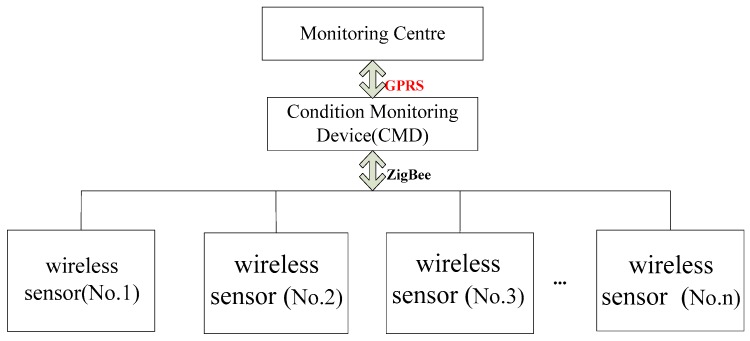
The functional blocks of an online monitoring system for conductor galloping of transmission lines.

The online monitoring technology of conductor galloping of transmission lines mainly comprises wireless sensor modules, a condition/state monitoring device (CMD), and condition monitoring center [12], as shown in Figure 2. The wireless sensor module is responsible for collecting the conductor galloping data and sending it to the CMD using a ZigBee network. The CMD is installed on the tower and receives data from the wireless sensor modules and transmits them to the monitoring center by a GPRS network. Expert software installed in the monitoring center can display line status information, diagnose running status, alarm, and forecast potential breakdowns.

## 2. Hardware Design of Wireless Sensor Module

**Figure 3:**
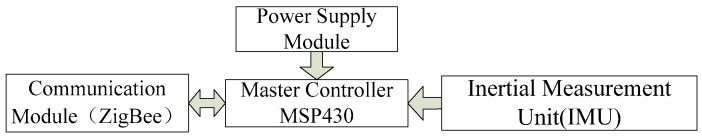
Design of the wireless sensor module.

The wireless sensor module, as shown in Figure 3, includes four units: an inertial measurement unit (IMU), a master controller, a power supply unit, and a wireless communication unit. Each unit will be briefly introduced as follows.

The inertial measurement unit measures the velocity, orientation, and gravitational forces of a point on the conductor, using a combination of accelerometers and gyroscopes, and sometimes also magnetometers. The inertial measurement unit consists of a three-axis acceleration sensor, a single-axis gyroscope and a dual-axis gyroscope. The three-axis ADXL330 acceleration sensor produced by Analog Devices, Inc. (ADI Company, Norwood, MA, USA) is employed in this work to measure the accelerations along the X-, Y-, and Z-axes. A single-axis Lpr530al gyroscope and a dual-axis Ly530alh gyroscope from the ST Company (Geneva, Switzerland) were chosen. They all have built-in signal conditioning circuits and output voltage signals. The Lpr530al is used for measuring the angle variations around the Z-axis, while Ly530alh measuring the angle variations around the X- and Y-axes. In practice, the sensitive axes of the sensors must be orthogonally installed to align the three axes along the coordinate frame of the measuring unit.

**Figure 4:**
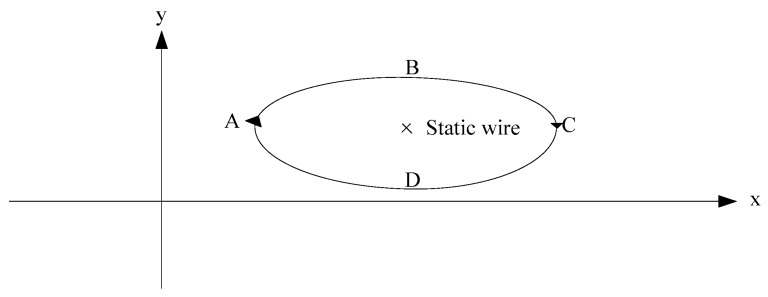
Elliptical trajectory of monitoring nodes.

Three axis accelerations and three axis angular rates under the carrier’s coordinate frame are collected by the IMU. Seen from the direction along the line, the trajectory of any point is similar to an ellipse when the conductor is galloping, with the elliptic plane perpendicular to the conductor, as shown in Figure 4.

**Figure 5:**
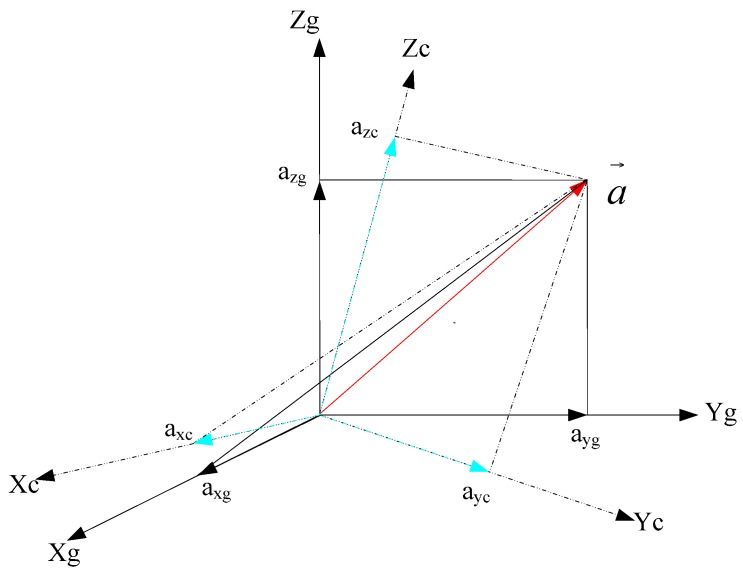
Transformation of coordinates.

**Figure 6:**
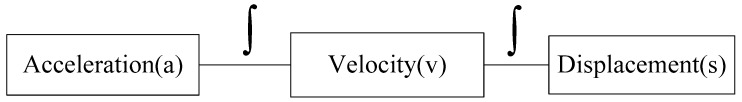
The diagram of changing acceleration to displacement.

To avoid errors caused by conductor twisting, all the characteristic quantities are calculated with respect to the geographical coordinate frame, that is to say, the accelerations (i.e., *a_xc_*, *a_y_*_c_ and *a_zc_*) with respect to the carrier’s coordinate frame are transformed into accelerations (i.e., *a_xg_*, *a_y_*_g_ and *a_zg_*) with respect to the geographical coordinate frame before calculation (as shown in Figure 5) using the following relationships:

(1) $$\left[\begin{array}{c} a_{xg}\\ a_{yg}\\ a_{zg} \end{array}\right]=[\begin{array}{ccc} c_{a_{xg},a_{xc}} & c_{a_{xg},a_{xc}} & c_{a_{xg},a_{xc}}\\ c_{a_{xg},a_{xc}} & c_{a_{xg},a_{xc}} & c_{a_{xg},a_{xc}}\\ c_{a_{xg},a_{xc}} & c_{a_{xg},a_{xc}} & c_{a_{xg},a_{xc}} \end{array}]\left[\begin{array}{c} a_{xc}\\ a_{yc}\\ a_{zc} \end{array}\right]=C\left[\begin{array}{c} a_{xc}\\ a_{yc}\\ a_{zc} \end{array}\right]$$

where *C* is referred to as the attitude matrix, which is commonly used for an attitude descriptor. Then the galloping displacements are gained through two integral operations, as illustrated in Figure 6.

Let’s take the X direction for example. Integrating the acceleration yields the velocity:

(2) $$v(t)-v(t_{0})=\int_{t_{0}}^{t}a_{xg}(t)dt$$

and integrating the obtained velocity yields the displacement:

(3) $$x(t)-x(t_{0})=\int_{t_{0}}^{t}v(t)dt$$

Velocity and displacement are calculated with the following formula:

(4) $$v(t)=v(t_{0})+\int_{t_{0}}^{t}a_{xg}(t)dt$$

(5) $$s_{x}(t)=x(t_{0})+\int_{t_{0}}^{t}v(t)dt$$

in which *a_xg_*(*t*) is the acceleration at time *t* (m/s^2^); *v*(*t*) is the velocity at time *t* (m/s); *v*(*t*_0_) is the initial velocity (m/s); and *s_x_*(*t*) is the displacement at time *t* (m).

**Figure 7:**
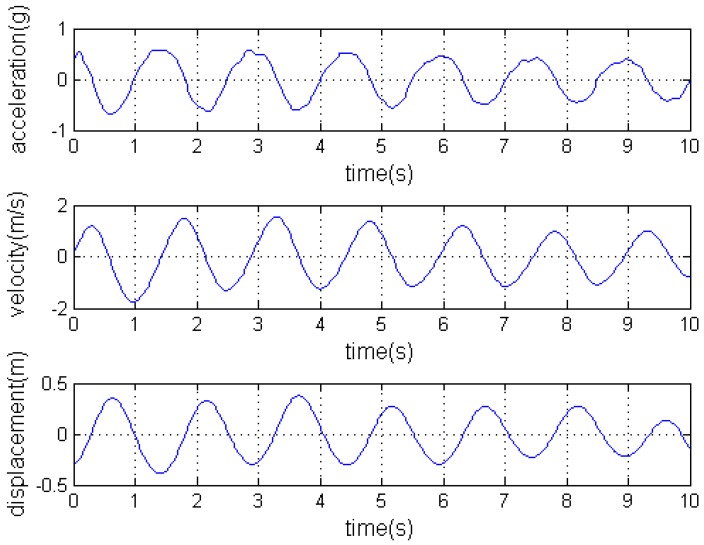
The plots of changing acceleration to displacement.

Similarly, the Y and Z direction displacements *s_y_*(*t*), *s_z_*(*t*) can be calculated and the conductor trajectory could be fit according to (*s_x_*(*t*), *s_y_*(*t*), *s_z_*(*t*)). Figure 7 shows the plots of changing acceleration into displacement.

The communication unit utilizes a short-distance ZigBee network, which is implemented by a CC2430 at 2.4 GHz radio frequency. A MSP430F247 microprocessor (TI company, Dallas, TX, USA) is selected as the master controller. Furthermore, considering that the wireless sensor module is installed on the conductor, the power supply module is designed through an open-loop transformer installed on the conductor. The current obtained by mutual inductance is then rectified, filtered, and regulated to form stable output voltage. The CMD receives the timing command after the wireless sensor module is powered on and sends a response to the sensors if the timing succeeds. Then the sensors begin to collect motion states of the conductor and send the results to the CMD. If the value exceeds the set threshold of the monitoring centre, the CMD will trigger an alarm.

### 2.1. Structure Design of Wireless Sensor Module

To avoid corona discharge and improve the aerodynamic characteristics, the wireless sensor shell has a smooth spherical structure made from aluminum. The shell was designed to be waterproof without affecting its normal operation. 

**Figure 8:**
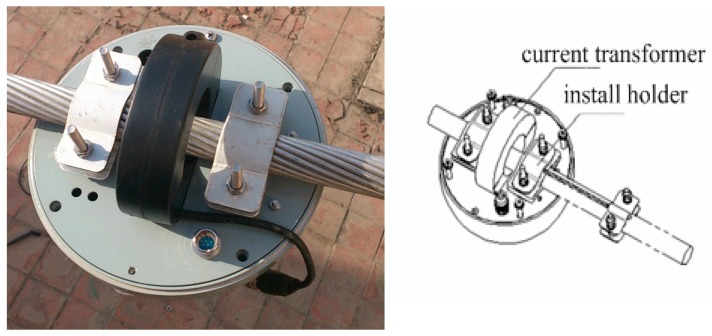
Structure design of the wireless sensor module.

The current transformer is embedded into a ring-type structure whose width is 27 mm, outer diameter is 150 mm and inner diameter is 55 mm. The sensor module can easily be installed and uninstalled, as shown in Figure 8. Through running on site, the wireless sensor module can meet the requirements of HV electrical characteristics and security.

### 2.2. Localization Algorithm of Wireless Sensor Module

The conductor torsion in the conductor galloping process is difficult to avoid, which causes the acceleration values collected at different times to not be in a same coordinate frame, so the key to realize the software is the calculation of the attitude matrix [17]. The sensor chooses the carrier coordinate frame as the reference system of a measured value, and the geographic coordinate frame as the transformed uniform reference system. The conversion between the carrier coordinate frame and the geographic coordinate frame is completed by the attitude matrix, built on the theory of a rigid body rotating about a fixed point in mechanics, in which the common methods describing the relationship of motion coordinate frame and reference coordinate frame are Euler angles, quaternion and direction cosine. However, due to the linearity and no singularity of differential equations, no trigonometrics in the integral program (as opposed to Euler angles), and the fewer parameters (relative to the direction cosine), the quaternion is chosen as the premier application method.

According to Euler’s rotation theorem, in three-dimensional space, any displacement of a rigid body such that a point on the rigid body remains fixed, can be represented by a single rotation about some axis that runs through the fixed point. The axis of rotation is known as the Euler axis denoted as $\vec{n}$, and the rotation angle is called the Euler angle denoted by *ζ* (Rodrigues-Hamilton parameters). In a reference system whose three unit vectors are $\vec{i},\;\vec{j}$ and $\vec{k}$, when a vector $Z=x\vec{i}+y\vec{j}+z\vec{k}$ rotates an angle around the instantaneous axis, the new coordinates can be calculated as:

(6) $$D=x_{1}\vec{i}+y_{1}\vec{j}+z_{1}\vec{k}\;{D=C}^{-1}ZC$$

where *Z* is the carriers coordinate frame, and *C* is the geographic coordinate frame:

(7) $$C=\cos\frac{1}{2}\zeta +\vec{n}\sin\frac{1}{2}\zeta$$

Equation (7) can be rewritten as a quaternion:

(8) $$C=\cos\frac{1}{2}\zeta +n_{x}\vec{i}\sin\frac{1}{2}\zeta +n_{y}\vec{j}\sin\frac{1}{2}\zeta +n_{z}\vec{k}\sin\frac{1}{2}\zeta$$

By denoting $c_{1}=\cos\frac{1}{2}\zeta ,\;c_{2}=n_{x}\cdot \sin\frac{1}{2}\zeta ,\;c_{3}=n_{y}\cdot \sin\frac{1}{2}\zeta \;$ and $c_{4}=n_{z}\cdot \sin\frac{1}{2}\zeta ,$ Equation (3) can be simply expressed as:

(9) $$c_{1}+c_{2}\vec{i}+c_{3}\vec{j}+c_{4}\vec{k},$$

Substituting Equation (9) into (6) yields:

(10) $$D=(c_{1}-c_{2}\vec{i}-c_{3}\vec{j}-c_{4}\vec{k})(x\vec{i}+y\vec{j}+z\vec{k})\cdot C,$$

which can be rewritten in a matrix form as:

(11) $$\left[\begin{array}{c} x_{1}\\ y_{1}\\ z_{1} \end{array}\right]=\left[\begin{array}{ccc} {c_{1}}^{2}+{c_{2}}^{2}-{c_{3}}^{2}-{c_{4}}^{2} & 2(c_{2}c_{3}{+c}_{1}c_{4}) & 2(c_{2}c_{4}-c_{1}c_{3})\\ 2(c_{2}c_{3}-c_{1}c_{4}) & {c_{1}}^{2}+{c_{3}}^{2}-{c_{2}}^{2}-{c_{4}}^{2} & 2(c_{3}c_{4}{+c}_{1}c_{2})\\ 2(c_{2}c_{4}{+c}_{1}c_{3}) & 2(c_{3}c_{4}-c_{1}c_{2}) & {c_{1}}^{2}+{c_{4}}^{2}-{c_{2}}^{2}-{c_{3}}^{2} \end{array}\right]\left[\begin{array}{c} x\\ y\\ z \end{array}\right]$$

From the above equation the attitude matrix can be known:

(12) $$\left[\begin{array}{ccc} {c_{1}}^{2}+{c_{2}}^{2}-{c_{3}}^{2}-{c_{4}}^{2} & 2(c_{2}c_{3}{+c}_{1}c_{4}) & 2(c_{2}c_{4}-c_{1}c_{3})\\ 2(c_{2}c_{3}-c_{1}c_{4}) & {c_{1}}^{2}+{c_{3}}^{2}-{c_{2}}^{2}-{c_{4}}^{2} & 2(c_{3}c_{4}{+c}_{1}c_{2})\\ 2(c_{2}c_{4}{+c}_{1}c_{3}) & 2(c_{3}c_{4}-c_{1}c_{2}) & {c_{1}}^{2}+{c_{4}}^{2}-{c_{2}}^{2}-{c_{3}}^{2} \end{array}\right],$$

From the above equation, we know that as long as the four elements c_1_, c_2_, c_3_ and c_4_ are obtained, and the attitude matrix can be derived, which can then be used to realize the conversion from the carrier’s coordinate frame to the geographic coordinate frame. What’s more, the angular rate w and quaternion *C* have the following relationship in quaternions:

(13) $$C^{\prime }=\frac{1}{2}A\cdot w$$

In the above formula, *w* is the angular rate matrix. Substituting the quaternion and angular rate component into the above equation, the following matrix form can be obtained:

(14) $$\left[\begin{array}{c} {c_{1}}^{\prime }\\ {c_{2}}^{\prime }\\ {c_{3}}^{\prime }\\ {c_{4}}^{\prime } \end{array}\right]=\frac{1}{2}\left[\begin{array}{cccc} c_{1} & -c_{2} & -c_{3} & -c_{4}\\ c_{2} & c_{1} & -c_{4} & c_{3}\\ c_{3} & c_{4} & c_{1} & -c_{2}\\ c_{4} & -c_{3} & c_{2} & c_{1} \end{array}\right]\left[\begin{array}{c} 0\\ w_{x}\\ w_{y}\\ w_{z} \end{array}\right]=\frac{1}{2}\left[\begin{array}{cccc} 0 & -w_{x} & -w_{y} & -w_{z}\\ w_{x} & 0 & w_{z} & -w_{y}\\ w_{y} & -w_{z} & 0 & w_{x}\\ w_{z} & w_{y} & -w_{x} & 0 \end{array}\right]\left[\begin{array}{c} c_{1}\\ c_{2}\\ c_{3}\\ c_{4} \end{array}\right]$$

*C* can be obtained when the above equation is solved. There are many analytical methods for solving differential equations. Commonly used methods include the Euler method, second-order Runge-Kutta method, fourth-order Runge-Kutta method, etc. Through a comparative analysis, the fourth-order Runge-Kutta method that produces smaller errors was adopted in this work. Combined with the fourth-order Runge-Kutta classic formula of numerical analysis, the simplified formula of Equation (9) is given as:

(15) $$\left\{ \begin{array}{l} C_{n+1}=C_{n}+\frac{h}{6}(k_{1}+2k_{2}+2k_{3}+k_{4})\\ k_{1}=\frac{1}{2}w\cdot C_{n}\\ k_{2}=\frac{1}{2}w\cdot (C_{n}+\frac{h}{2}\cdot k_{1})\\ k_{3}=\frac{1}{2}w\cdot (C_{n}+\frac{h}{2}\cdot k_{2})\\ k_{4}=\frac{1}{2}w\cdot (C_{n}+h\cdot k_{3}) \end{array}\right.$$

In the above equation, *h* is the sampling interval, $W=\left[\begin{array}{cccc} 0 & -w_{x} & -w_{y} & -w_{z}\\ w_{x} & 0 & w_{z} & -w_{y}\\ w_{y} & -w_{z} & 0 & w_{x}\\ w_{z} & w_{y} & -w_{x} & 0 \end{array}\right]$, $C_{n}$ = ${\left[\begin{array}{c} c_{1}\\ c_{2}\\ c_{3}\\ c_{4} \end{array}\right]}_{n}$, and $k_{n}=\left[\begin{array}{c} k_{n0}\\ k_{n1}\\ k_{n2}\\ k_{n3} \end{array}\right]$. Then the first item is derived as:

(16) $${\left[\begin{array}{c} c_{1}\\ c_{2}\\ c_{3}\\ c_{4} \end{array}\right]}_{n+1}={\left[\begin{array}{c} c_{1}\\ c_{2}\\ c_{3}\\ c_{4} \end{array}\right]}_{n}+\frac{t}{6}\left[\begin{array}{c} k_{10}\\ k_{11}\\ k_{12}\\ k_{13} \end{array}\right]+2\left[\begin{array}{c} k_{20}\\ k_{21}\\ k_{22}\\ k_{23} \end{array}\right]+2\left[\begin{array}{c} k_{30}\\ k_{31}\\ k_{32}\\ k_{33} \end{array}\right]+\left[\begin{array}{c} k_{40}\\ k_{41}\\ k_{42}\\ k_{43} \end{array}\right]$$

*k_n_* can be solved according to the following three equations. This is the solution procedure of *C*, and then attitude matrix is obtained after *C* is directly substituted into Equation (10).

**Figure 9:**
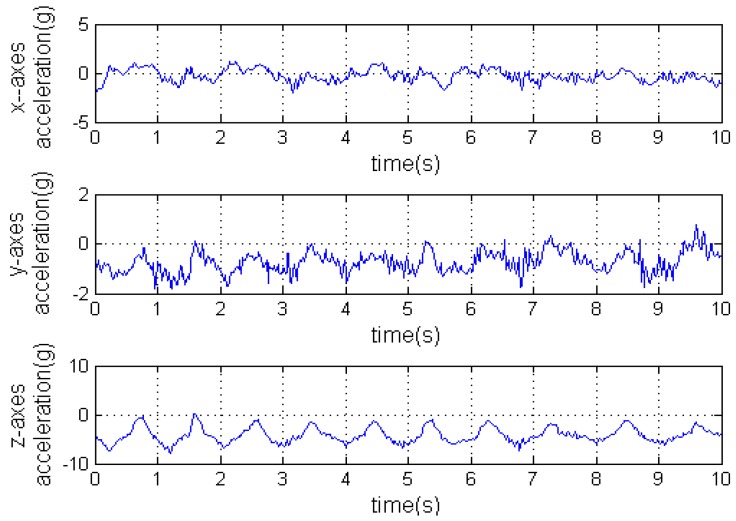
The accelerations with respect to the carrier’s coordinate frame.

**Figure 10:**
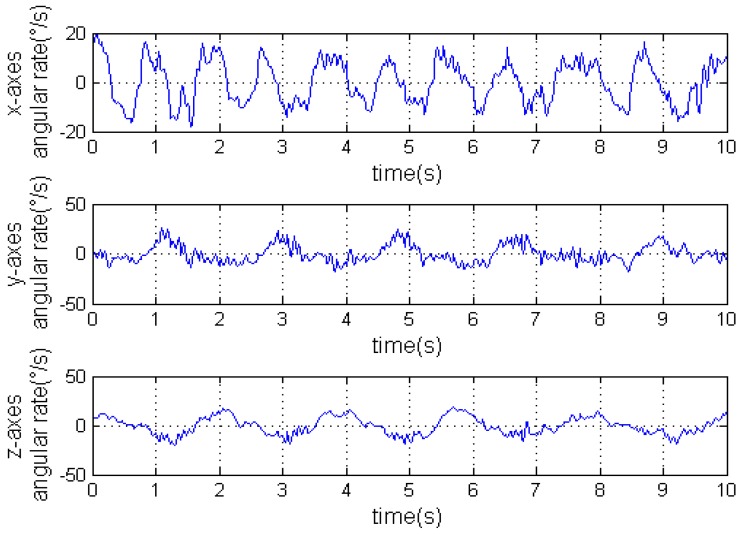
The angular rate.

**Figure 11:**
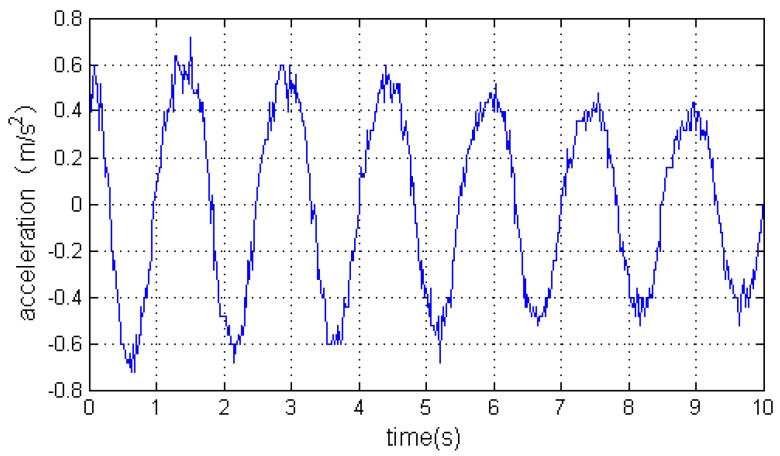
Y-axes accelerations with respect to the geographical coordinate frame.

Once *C* is worked out, we can get the accelerations with respect to the geographical coordinate frame using Equation (1). Figure 9 is the accelerations with respect to the carrier’s coordinate frame measured by the IMU. Figure 10 is the angular rates measured by the IMU. Finally, the accelerations with respect to the geographical coordinate frame on the Y axis can be seen in Figure 11.

In this paper, the conductor galloping trajectory is obtained using the Bezier curve fitting method through the displacements measured by the sensors installed on the conductor. Suppose that *n* galloping sensors were installed on a span. For a Bezier curve, *n* control points are obtained, with each sensor position given as:

(17) $$s_{k}=(s_{kx},s_{ky},s_{kz})\;k=0,1,...,n-1$$

The conductor galloping wave *S*(*u*) can be written as:

(18) $$S(u)=[\begin{array}{ccc} x\left(u\right) & y\left(u\right) & z\left(u\right) \end{array}]=\sum_{k=0}^{n-1}\left[\begin{array}{ccc} s_{kx} & s_{ky} & s_{kz} \end{array}\right]BEZ_{k,n-1}(u)$$

where $BEZ_{k,n-1}\left(u\right)=C\left(n-1,k\right)u^{k}{\left(1-u\right)}^{n-1-k}=C_{n-1}^{k}u^{k}{\left(1-u\right)}^{n-1-k}$, and *u* is the ratio of installation location and distance between towers:

(19) $$u=\frac{d_{i}}{d},$$

where *d_i_* is the horizontal distance between the *i-*th sensor and the small side of tower and *d* is the span.

### 2.3. Other Considerations

**Figure 12:**
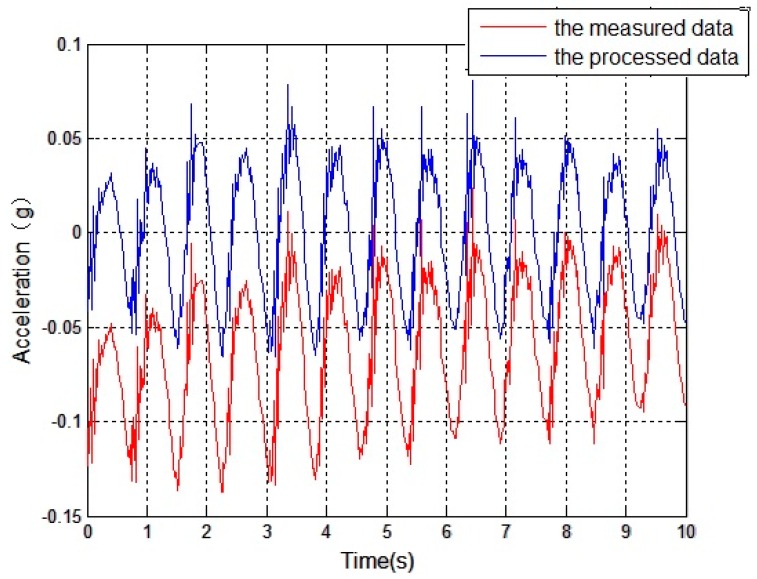
Comparison of the signals before and after being processed.

During the data collection process of the sensors, due to temperature drift and outside interference, the received signals often contain DC components and trend items, whose existence have a large influence on the subsequent integral operations, and may even yield distortions. Therefore, the mean method and the least squares method are used to remove the DC components and the trend items. Figure 12 compares a set of measured data, of which the data contain the DC component and the trend items are represented by the red line, and the processed data are represented by the blue line. This shows that the processing effect is good.

**Figure 13:**
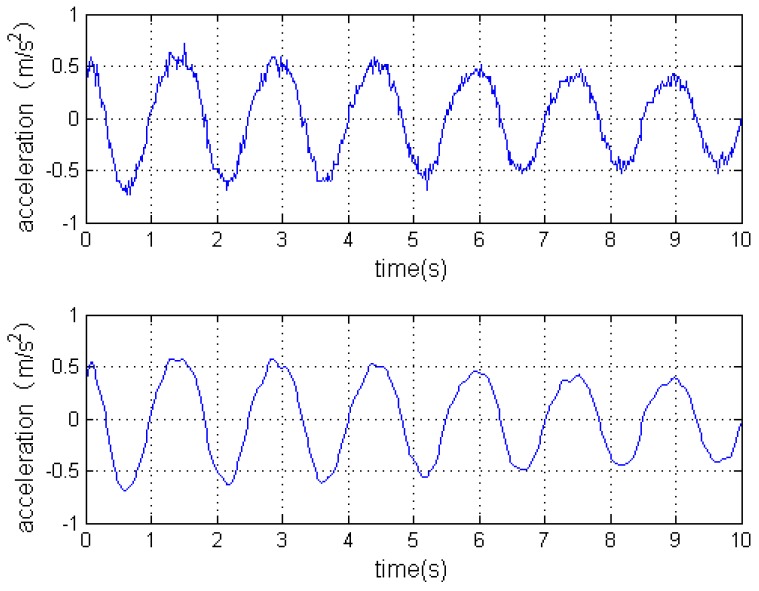
Comparison of the signals before and after low-pass filtering.

Selecting acceleration data of one direction in the reciprocating trial in one period as an example, Figure 13 illustrates the effect of low-pass filtering. The first plot is the waveform simulation of the original measured acceleration, and the second plot shows the signal after low pass filtering. The original measured acceleration signal showing high frequency interference get smoother after low-pass filtering, which makes the acquired data more accurate and easier to use for subsequent processing.

When the frequency of galloping ranges from 0.1 to 3 Hz, it may cause the measured amplitude to be inconsistent with the actual data, and distortions can even occur when the cut-off frequency is set too low, and the interference signal will not be filtered out well if set too high. In this paper, the cut-off frequency is set in the software at 5 Hz and the effect is good.

## 3. Experimental Test and Analysis

### 3.1. Accuracy Test of the Single Sensor Based on Test Platform

**Figure 14:**
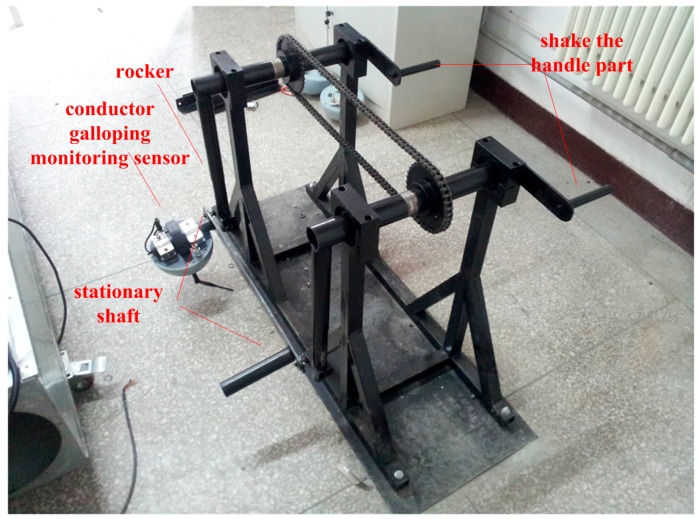
Accuracy test setup.

**Figure 15:**
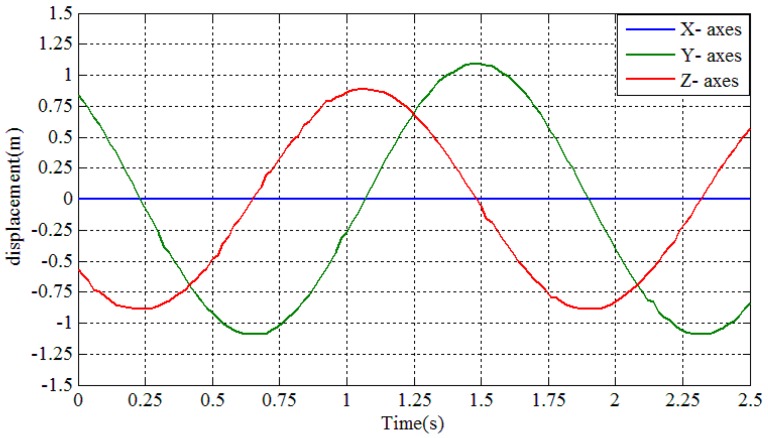
Collected data of the accuracy test.

A test platform, as shown in Figure 14, was built to test the measuring accuracy of the proposed conductor galloping sensor. The conductor galloping sensor was installed on the test equipment with the X-axis parallel to the fixed axis and the Z-axis perpendicular to the ground. As the shaft performs a reciprocating movement, the accelerations and angular rate measured by the conductor galloping monitoring sensor were transmitted to a computer with software installed especially for the test installed by a ZigBee network. After being processed, the results of the displacements along the X-, Y- and Z-axes are shown in Figure 15. In addition, the known biggest swing amplitude is 1 m.

The test results show that the maximum displacements along the Y- and Z- axis are 1.095 m and 0.889 m, respectively, which agree well with the actual conductor movement.

### 3.2. Accuracy Test of the Single Sensor Based on Conductor

**Figure 16:**
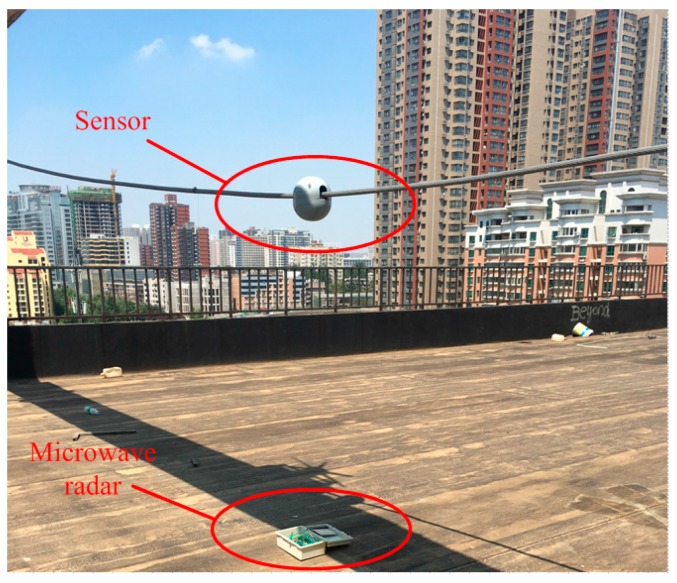
Experiment setup of a single sensor.

An experiment setup was built to test the feasibility of the monitoring sensors, as illustrated in Figure 16. One end of the conductor is fixed, and the other end is fixed on the handle of a machine. The machine can excite the conductor to perform an elliptical motion in one direction by driving the handle vibration. A microwave radar meter is used as benchmark for measuring the length of the semi-major axis and semi-minor axis [18]. The collected data of the tri-axial accelerations and angular rates are sent to a computer with a software installed especially for the test using ZigBee. Then the relative displacement curves were obtained through fitting. 

**Figure 17:**
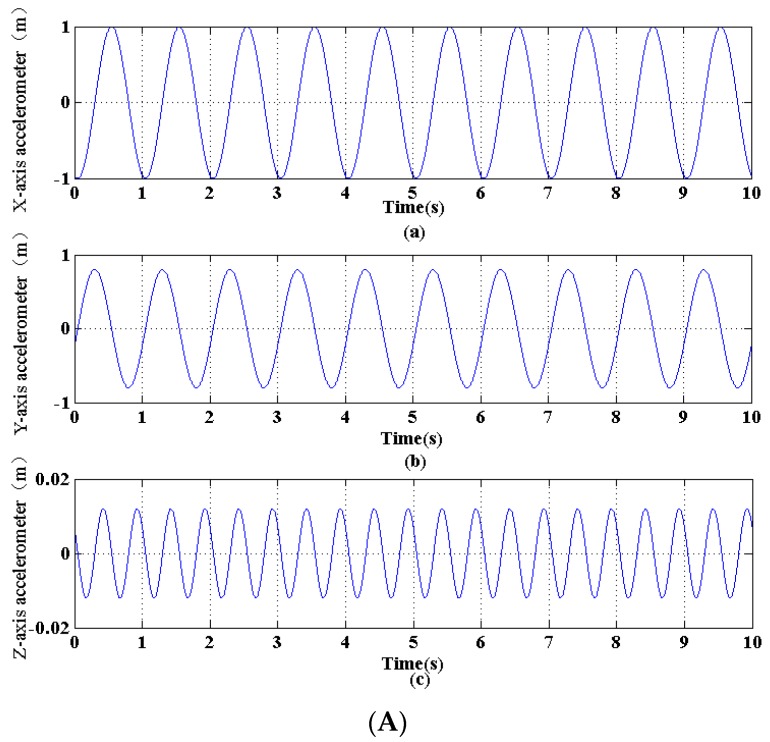

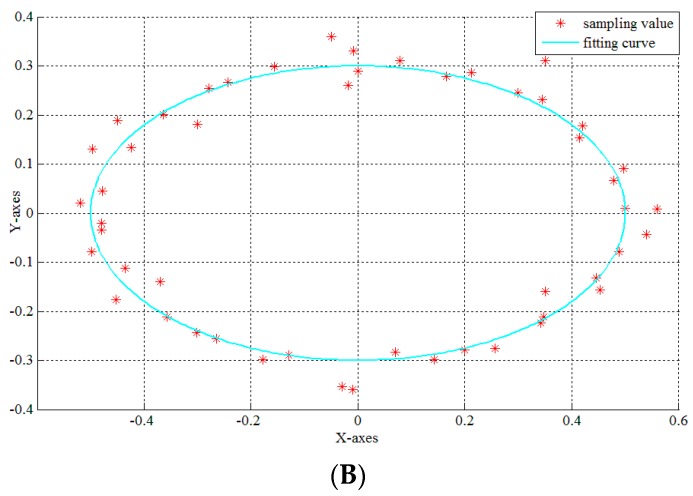
The displacement fitting at a single monitoring point: (**A**) Motion displacements of a single node; (**B**) Trajectory of a single point in a cycle (view from the Z-axis).

In the experiments, the semi-major axis of elliptical motion ranges from about 0.5 m to about 2 m, and the semi-minor axis ranges from about 0.3 m to about 1.3 m. Figure 17 shows how to get elliptical motion parameters according to a set condition where the length of the semi-major axis is 0.5 m and the length of the semi-minor axis is 0.3 m. Then many different motions were measured by both the designed sensor and the radar meter, and the results are shown in Table 1. It can be seen that the maximum relative error of the semi-major axis is 8.11%, and the maximum relative error of the semi-minor axis is 8.35%.

### 3.3. Application of the Single Sensor

**Figure 18:**
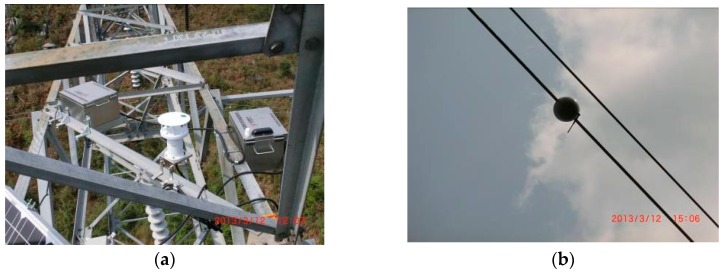
Field installation of the wireless sensor module. (**a**) CMD; (**b**) wireless sensor.

The wireless sensor module has been tested in the Guizhou power grid. Three sensors were installed on a conductor spanning 734 m. The field installation is shown in Figure 18.

**Figure 19:**
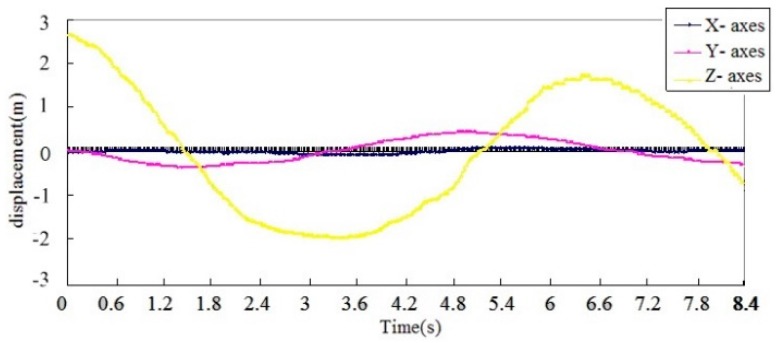
The runtime data curve.

Figure 19 plots the data obtained by the wireless sensor module whose ID is 2 describing the conductor galloping status in a period on 28 May 2013. It can be clearly seen from this figure that the displacement along the X-axis is the smallest, while the displacement along the Z-axis is the largest. The results agree well with those recorded by the video camera.

## 4. Conclusions

With the higher state grid scale, monitoring technologies for transmission lines are becoming more and more important. A kind of wireless sensor module for monitoring conductor galloping of transmission lines has been proposed and realized in this work. The feasibility and accuracy of wireless sensor module were verified on the corresponding test platform. In general, the proposed wireless sensor module appears to be very useful for monitoring galloping. Further work will focus on how many sensors should be installed between two consecutive towers to achieve the optimal monitoring performance.

## Figures and Tables

**Table 1:** Experimental data of the elliptical motion.

Semi-Major Axis Measured by Sensor (m)	Semi-Minor Axis Measured by Sensor (m)	Semi-Major Axis by Radar (m)	Semi-Minor Axis by Radar (m)	Relative Error of Semi-Major Axis	Relative Error of Semi-Minor Axis
0.473	0.267	0.497	0.284	4.83%	5.99%
0.492	0.295	0.513	0.311	4.09%	5.14%
0.906	0.593	0.986	0.623	8.11%	4.82%
1.029	0.678	1.114	0.712	7.63%	4.78%
1.431	0.757	1.538	0.826	6.96%	8.35%
1.483	0.874	1.585	0.921	6.44%	5.10%
2.026	1.215	2.109	1.318	3.94%	7.81%
2.156	1.281	2.253	1.396	4.31%	8.24%
